# Targeting bacterial adherence inhibits multidrug-resistant *Pseudomonas aeruginosa* infection following burn injury

**DOI:** 10.1038/srep39341

**Published:** 2016-12-20

**Authors:** Ryan M. Huebinger, Daniel H. Stones, Marcela de Souza Santos, Deborah L. Carlson, Juquan Song, Diana Pereira Vaz, Emma Keen, Steven E. Wolf, Kim Orth, Anne Marie Krachler

**Affiliations:** 1 Department of Surgery, University of Texas Southwestern Medical Center, Dallas, TX, USA; 2 Institute of Microbiology and Infection, School of Biosciences, University of Birmingham, Birmingham B15 2TT, UK; 3 Department of Molecular Biology, University of Texas Southwestern Medical Center, Dallas, TX, USA; 4 Department of Biochemistry, University of Texas Southwestern Medical Center, Dallas, TX, USA; 5 Howard Hughes Medical Institute, University of Texas Southwestern Medical Center, Dallas, TX, USA; 6 Department of Microbiology and Molecular Genetics, University of Texas McGovern Medical School at Houston, Houston, TX, 77030, USA

## Abstract

Classical antimicrobial drugs target proliferation and therefore place microbes under extreme selective pressure to evolve resistance. Alternative drugs that target bacterial virulence without impacting survival directly offer an attractive solution to this problem, but to date few such molecules have been discovered. We previously discovered a widespread group of bacterial adhesins, termed Multivalent Adhesion Molecules (MAMs) that are essential for initial binding of bacteria to host tissues and virulence. Thus, targeting MAM-based adherence is a promising strategy for displacing pathogens from host tissues and inhibiting infection. Here, we show that topical application of polymeric microbeads functionalized with the adhesin MAM7 to a burn infected with multidrug-resistant *Pseudomonas aeruginosa* substantially decreased bacterial loads in the wound and prevented the spread of the infection into adjacent tissues. As a consequence, the application of this adhesion inhibitor allowed for vascularization and wound healing, and maintained local and systemic inflammatory responses to the burn. We propose that MAM7-functionalized microbeads can be used as a topical treatment, to reduce bacterial attachment and hence prevent bacterial colonization and infection of wounds. As adhesion is not required for microbial survival, this anti-infective strategy has the potential to treat multidrug-resistant infections and limit the emergence of drug-resistant pathogens.

Burns contribute to a major fraction of trauma injuries and many trauma-associated fatalities occur in burn patients. In the United States alone, there are over a million burn injuries, leading to 100,000 hospitalizations annually^1^. As the skin is the body’s first and major defense mechanism against microbial intrusion, patients enduring burn trauma are particularly susceptible to infections. Immediately following injury, the body responds with systemic immunosuppression^2^,^3^. Up to 75% of the mortality in burn patients is associated with an infection, particularly in patients suffering severe burns of 40% total body surface area or above.

Sepsis and graft rejection are dangerous clinical outcomes for patients with burn wound infections. Although immediate care for burn patients has improved due to treatments such as hemodynamic stabilization^4^,^5^, more strategies to minimize burn wound infections are urgently needed to reduce patients’ morbidity and mortality. With the increase in antibiotic resistant bacteria and the narrowing pipeline for developing new antimicrobials, the challenges for treating infection are ever more difficult^6^.

Bacterial infections in burn patients are one of the most common causes of mortality. The Gram-negative bacterium *Pseudomonas aeruginosa* is frequently associated with hospital acquired infections^7^,^8^ and is found in approximately 33% of all burn wounds and in 59% of extensive burns^1^. Due to drug efflux mechanisms and genetically acquired drug resistance, *P. aeruginosa* is extremely antibiotic tolerant^9^. These characteristics have led to the rise of multidrug-resistant isolates that are observed in burn patients and transferred between patients through healthcare personnel or hospital equipment^10^,^11^. The intrinsic modes of bacterial defense allow for their persistence in clinical settings and current treatments that use more antibiotics are resulting in increasing antimicrobial resistance.

It is now globally recognized that there is an urgent need for new antimicrobial drugs. However, antimicrobials that prevent replication exert strong selective pressure, leading to the rapid acquisition of resistance and thus new antimicrobials that target microbial virulence, rather than growth, are an attractive therapeutic option. Finally, these new drugs should allow for normal wound healing in the patient^12^,^13^. Although anti-virulence drugs have been an emerging concept for the last 50 years, to date very few have been tested for their efficacy in infection models^14^,^15^ and none are capable of targeting a broad spectrum of pathogens^13^. Here we provide evidence that microbeads functionalized with MAM7 adhesin meet the criteria for such a “new antimicrobial”.

MAM7 is a bacterial outer membrane protein that promotes host cell adhesion by a wide range of Gram-negative bacteria^16^. Previously, *in vitro* studies demonstrated that recombinant MAM7 prevents Gram-negative pathogens and methicillin resistant *Staphylococcus aureus* from binding to host cells, thereby inhibiting subsequent infection and cytotoxicity. We also reported that the binding avidity, and thus competitive effect of MAM7 could be drastically increased by multivalent surface display^17^,^18^,^19^,^20^. To our knowledge, MAM7 is the first reported virulence-targeting compound with a broad spectrum activity. Preliminary cell culture studies showed that treatment with recombinant MAM7 does not interfere with cellular processes involved in wound healing, such as proliferation, extracellular matrix deposition, and migration^17^. Based on these promising traits, we set out to test the efficacy of MAM7 as a potential inhibitor of multidrug-resistant *P. aeruginosa* in a murine burn and excision infection model. We found that microbeads functionalized with MAM7 adhesin act as an ideal antimicrobial agent that could potentially bring down bacterial colonization in a burn patient to a sub-lethal level.

## Results

### A bacteriomimetic inhibitor based on the adhesin MAM7 decreases bacterial burden on burn wounds

**Figure 1:**
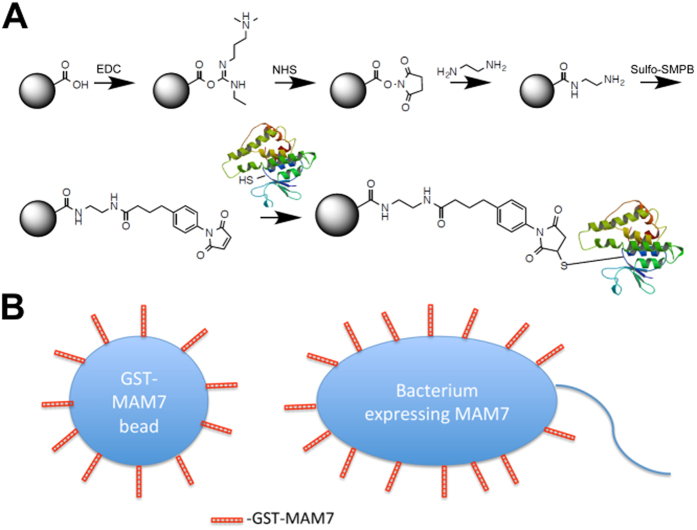
Schematic of inhibitor synthesis. **(A)** Carboxy-functionalized polystyrene micro-beads of 1 μm diameter are activated using EDC/NHS, and covalently coupled to GST (control beads) or GST-MAM7 (inhibitor beads) using Sulfo-SMPB. This results in directional coupling of recombinant proteins to the bead surface via the cysteine-containing GST domain. **(B)** Schematic of GST-MAM7 bead mimicry of bacterial MAM7 presentation.

The adhesin MAM7 is an outer membrane protein that is widely expressed by Gram-negative pathogens. It consists of seven protein domain repeats that are responsible for attachment to host cells during an infection. A recombinant fragment of MAM7 containing these repeats is expressed as a Glutathione-S-Transferase (GST) fusion protein, GST-MAM7. Purified, recombinant GST-MAM7 is chemically coupled to a micron-sized, spherical polystyrene scaffold and this composition provides a mimic for its endogenous presentation on the surface of a bacterium (Fig. 1). Multivalent surface display significantly enhances binding avidity and thus, the material’s competitive effect against pathogens (Hawley *et al*.^17^). GST-MAM7 functionalized beads and control microbeads functionalized with only GST were synthesized on a milligram scale and tested in a quality control inhibition assay using an *in vitro* infection model as previously described^17^,^18^,^19^,^20^.

**Figure 2:**
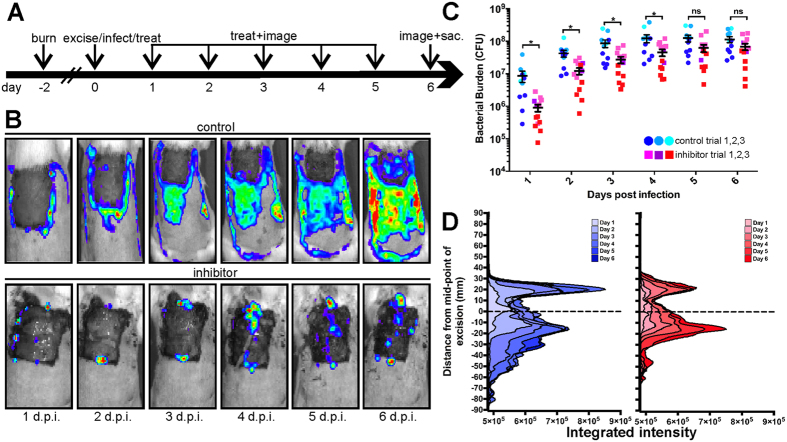
Treatment of excised burn wounds with a GST-MAM beads decreases bacterial burden and spatially constrains the spread of infection. (**A**) Schematic of infection and dosing schedule. (**B**) Representative bioluminescence images of control bead treated (upper row) or MAM7 inhibitor treated (bottom row) *P. aeruginosa* infected excisions over 6 days after infection (d.p.i.). (**C**) Quantification of bacterial loads in control bead-treated (shades of blue) and inhibitor treated (shades of red) animals using IVIS biophotonic imaging. Different shading indicates three sets of independent trials. Data for individual animals, means ± s.e.m. for each treatment group are depicted. Analysis of variance (ANOVA), followed by Tukey’s post hoc test, was used to test for significance. (*) indicates p ≤ 0.05, ns, not significant. (**D**) Quantitative analysis of the spread of infection to adjacent tissues.

**Figure 3:**
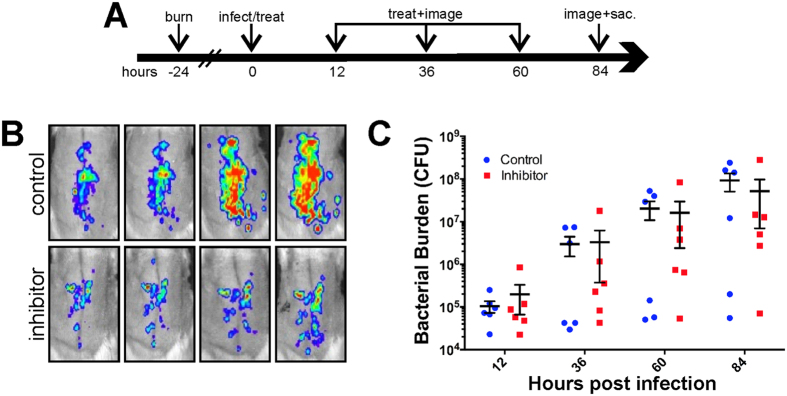
Inhibition of *P. aeruginosa* in unexcised burns. Schematic of infection and dosage regimen **(A)**. Representative bioluminescence images of MAM7 inhibitor treated (bottom row) and control bead treated (upper row) rats infected with *P. aeruginosa* **(B)**. Quantification of bacterial burden by IVIS photon flux analysis **(C)**.

To assess whether GST-MAM7 beads could decrease the bacterial load in burns, we used a burn and excision infection rat model. For severe burns, early excision, followed by application of wound dressings and/or grafting is currently the standard of care^1^. Early surgical excision to remove necrotic tissue within the first few days following injury is routinely used in treatment of extensive burns, and has significantly contributed to decreased occurrence of burn wound sepsis and improved survival^21^,^22^. For this study, dorsal burn wounds covering 40% of the total body surface area were induced onto the back of adult rats and two days after injury, eschar tissue was surgically excised (Fig. 2A)^23^. Excision prior to treatment with the MAM7 inhibitor increased the treatment efficacy, compared to unexcised, necrotic burns (Fig. 3). Coincidently, on day two, a suspension of 5·10^6^ CFU of *P. aeruginosa* Xen5, a bioluminescent derivative of a multidrug-resistant septicemia blood isolate^24^, were introduced into the excision. Suspensions containing 3·10^8^ GST beads or GST-MAM7 beads in saline were applied topically to the wound bed at the same time and, then, applied every 24 hours after excision. The bacterial loads of the infection were monitored by biophotonic imaging over 6 days (Fig. 2A).

In the infection that was treated with control GST beads, the bacteria were initially constrained to the edge of the surgical excision, but gradually spread across the entire burn area over the course of 6 days (Fig. 2B). By contrast, in wounds treated with GST-MAM7 beads, the bacteria remained on the edge of the wound (Fig. 2B). Over the first 24 hours, GST-MAM7 bead treatment reduced the bacterial burden below the initial inoculum and, then, remained lower than the control treatment for an additional three days. In control treated animals, the bacterial burden increased approximately 10-fold over the course of the 6 day experiment (Fig. 2C). Treatment with the MAM7 inhibitor beads significantly decreased bacterial colonization of the burn for four days following infection when compared to mock treatment, although leveled off towards the end of the experiment. These studies demonstrate that fresh wounds can be treated with GST-MAM7 functionalized beads as an early intervention and the bacterial load will be reduced in the excision and surrounding burnt area (Fig. 2).

### Treatment of excised burn wounds with GST-MAM beads decreases bacterial spread

We considered the possibility that the anti-adhesion activity of MAM7 may simply displace burn-colonizing bacteria to adjacent tissue sites. To address this, we used biophotonic imaging to follow the spread of the infection over the wounded area (Fig. 2D; the peaks represent bacterial burden across the wound). In the control animals treated with GST-beads, we observed a gradual spread of bacteria (light blue to dark blue) over the wound during the course of the 6 day experiment. By contrast, in the GST-MAM7 bead treated animals, the spread and amount of bacterial colonization (pink to red) is substantially lower during the experimental time course. This demonstrates that GST-MAM7 beads not only lower the total bacterial burden in the wound, but spatially confine bacterial colonization and inhibit the spread of infection.

### Beads functionalized with MAM7 adhesin do not perturb angiogenesis or wound closure

**Figure 4:**
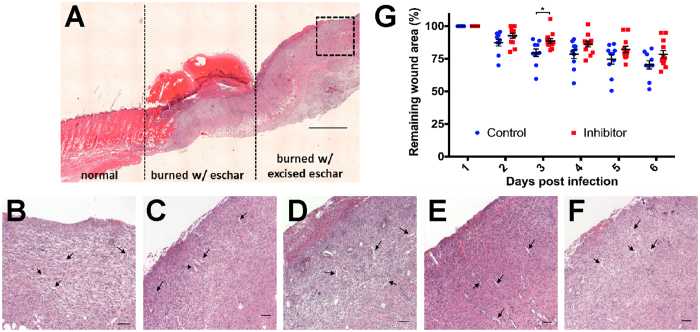
Angiogenesis and wound closure are unaffected by treatment with GST-MAM7 beads. (**A**) H&E-stained skin tissue sampled post-mortem (6 d.p.i.) depicting normal skin **(left section)**, burned skin with eschar **(middle section)**, and burned skin with excised eschar **(right section)**. Boxed area highlights region that was used to analyze vascularization in the following samples: **(B)** burned skin only, **(C)** burned skin with GST-beads, **(D)** burned skin with GST-MAM7-beads, **(E)** burned skin with GST-beads + *P. aeruginosa*, and **(F)** burned skin with GST-MAM7-beads + *P. aeruginosa*. Arrows point to blood vessels. Scale bar equals to 1000 μm in **(A)** and 100 μm in **(B–F)**. **(G)** Analysis of excision closure from excised, uninfected animals.

Antimicrobial treatments for wounds must not impair the natural wound healing process. Thus, to assess the impact of GST-MAM7 beads on vascularization and wound closure, we analyzed H&E sections of burned tissue on day 6 of the infection. In Fig. 4A, a section of skin containing a burn is shown in three parts: 1. unburned, normal tissue; 2. burned tissue with eschar intact; 3. burned tissue with the eschar excised. Analysis of an uninfected tissue sample from the burned tissue with the eschar excised shows normal granulation, vascularization and initiation of wound healing (Fig. 4A). When the GST-beads or GST-MAM7 beads are applied to the burned tissue with the eschar excised, similar granulation, vascularization and initiation of wound healing is observed (Fig. 4C and D). Therefore, the beads alone did not disrupt normal healing. Burned tissue with the eschar excised that was treated with *P. aeruginosa* and the GST-beads or GST-MAM7 beads also appeared to heal in a normal manner (Fig. 4E and F). Further quantitative analysis of wound closure revealed no significant differences in healing between GST-beads or GST-MAM7 beads treated groups, with excision areas decreasing by approximately 22 and 30%, respectively, over 6 days (Fig. 4G). Thus, MAM7 bead treatment does not impair normal burn wound healing.

### Normal neutrophil infiltration of burned skin is observed in GST-MAM7 bead treatment

**Figure 5:**
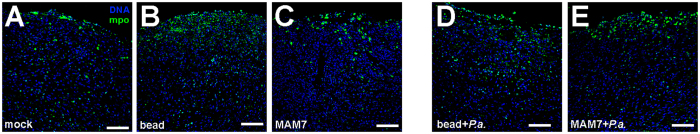
Neutrophil infiltration on burned skin is unimpaired by treatment with GST-MAM7 beads. Confocal micrographs of burned skin area as in Fig. 1A was used to analyze neutrophil infiltration for the following samples: **(A)** burned skin only, **(B)** burned skin with GST-beads, **(C)** burned skin with GST-MAM7-beads, **(D)** burned skin with GST-beads + *P. aeruginosa*, and **(E)** burned skin with GST-MAM7-beads + *P. aeruginosa*. Hoechst DNA (blue) and mpo staining for neutrophils (green). Scale bar equals to 150 μm. Samples were taken post-mortem (6 d.p.i.).

Severe burns generally elicit a strong inflammatory response, both locally and in the tissues adjacent to the burn^25^. A moderate pro-inflammatory response encourages wound healing, but an excessive inflammatory response leads to organ damage and dysfunction^26^. We therefore tested if application of control beads or adhesin beads to burns without and with *P. aeruginosa* would have an effect on local inflammation. Adjacent sections of tissues shown in Fig. 3 were used to assess neutrophil infiltration. In untreated animals, a normal migration of neutrophils moving toward the wounded surface area is observed (Fig. 5).

**Figure 6:**
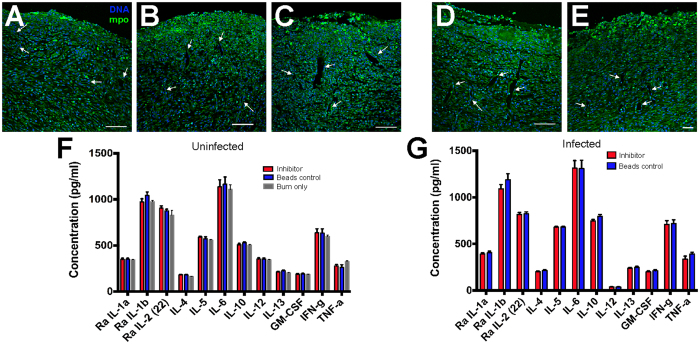
Neutrophil infiltration and vascularization of burned skin is unimpaired by GST-MAM7 beads. Confocal micrographs of burned skin (as in boxed area depicted in Fig. 1A) were used to analyze neutrophil infiltration (bright green fluorescent clumps) and vascularization (observed through green channel background) for the following samples: **(A)** burned skin only, **(B)** burned skin with GST-beads, **(C)** burned skin with GST-MAM7-beads, **(D)** burned skin with GST-beads + *P. aeruginosa*, and **(E)** burned skin with GST-MAM7-beads + *P. aeruginosa*. Arrows point to blood vessels. Scale bar equals to 150 μm. **(F)** Serum cytokine levels of burned only (grey), burned inhibitor (red) or burned control (blue) treated uninfected animals. **(G)** Serum cytokine levels of inhibitor (red) or control (blue) treated animals with *P. aeruginosa* infected burns. Data presented are means ± stdev (n = 6 for inhibitor and bead treated groups, n = 5 for burn only group, each sample measured in triplicate against cytokine standards). All samples were taken post-mortem (6 d.p.i.).

In addition, as observed with the green channel background, vascularization is occurring in the healing tissue (Fig. 6A). The application of GST beads or GST-MAM7 beads to uninfected or *P. aeruginosa* infected burned and excised wounds did not lead to a change near newly formed blood vessels (Fig. 6A–E) or neutrophil migration towards the wound (Figs 5A–E and 6A–E). Overall, the observation that GST-MAM7 does not hinder wound healing are consistent with our *in vitro* infection studies showing that, despite MAM7 binding to host surface receptors including fibronectin^27^, MAM7 inhibitor treatment does not interfere with processes intimately associated with wound healing, including proliferation and extracellular matrix formation^17^.

### Application of adhesin functionalized beads does not alter systemic inflammatory reponsens

Although wound healing was visually normal in adhesin-bead treated wounds, we considered the possibility that the application of these beads may provoke a systemic inflammatory response that could be deleterious to the host. We therefore tested levels of circulating serum cytokines in the test animals. However, in all cases animals that were untreated or treated with GST beads or GST-MAM7 beads expressed similar levels of circulating serum cytokines (Fig. 5F). In animals infected with *P. aeruginosa*, the induction of circulating serum cytokines was unaltered by application of MAM7 adhesin beads, compared to control beads (Fig. 5G). Therefore, the application of MAM7 inhibitor to *P. aeruginosa* infected wounds did not alter systemic inflammatory responses.

## Discussion

Taken together, our data demonstrate for the first time, the efficacy of a virulence-targeting antimicrobial compound with broad specificity in an *in vivo* model. Microbeads functionalized with a recombinant fragment of the adhesin MAM7 fulfill the requirements for new strategies to combat pathogens in a particularly challenging clinical setting: deep tissue burns. First, the MAM7-mimicking inhibitor competes with the pathogen for sites on the host to colonize and initiate an infection. The success of this competition depends on the availability of native receptors on the host cell surface (compare Figs 2 and 3). As this mechanism of inhibition targets host colonization, but is non-essential for the pathogen’s survival, there is likely less evolutionary selection for a mechanism of resistance. Second, the bacteriomimetic inhibitor does not have any deleterious impact on wound healing, making it an excellent candidate for future use on patients. Third, there is no enhanced inflammatory response to MAM7 inhibitor treatment. We rationalize this is because hosts (patients) are immunotolerant to MAM7-like molecules because they have been exposed to MAMs displayed by commensal bacteria throughout development^20^. Therefore, as expected, the host does not mount an altered local or systemic immune response to GST-MAM7 beads. The inflammatory response to the infection was not significantly elevated compared to burnt, uninfected animals. This is likely because of the limited potential of the *P. aeruginosa* strain used to invade host tissues and spread systemically. The absence of systemic spread was underpinned by the absence of systemic bioluminescence, even at high exposure settings, and by the absence of clinical signs of sepsis in all animals. Future work will address if this treatment is efficacious for the treatment of invasive infections as well.

The treatment with GST-MAM7 beads was highly efficacious, and decreased the bacterial burden significantly for four days following infection. This is likely due to the limited potential of displaced bacteria to grow and persist in the exudate, compared to bacterial in direct contact with the tissue. The beneficial effect of treatment for the infected host may be underestimated by measuring bacterial burden, due to the fact that bacteria displaced from the tissue are partially trapped in the wound exudate crust, and not completely removed from the wound, so that they still contribute to luminescence. Future work will attempt to better discriminate between directly attached bacteria, and bacteria which are displaced from the tissue but retained in the semi-solid exudate matrix covering the wound bed, by measuring luminescence before and after exudate removal. However, the pathogenic potential of displaced bacteria is significantly reduced compared to bound bacteria, since many virulence factors, such as transfer of effectors via type III secretion stems, rely on intimate contact between bacterium and host cell^16^,^28^. Although bacteria are not eradicated from the wound site by MAM7 beads, such a treatment creates a therapeutic window that slows the progress of bacterial colonization and infection, even under the high bacterial burden used in the study. An altered dosing schedule with higher concentrations and more frequent applications of GST-MAM7 beads over the first 24–48 hours following infection might reduce bacterial colonization further. Alternatively, this strategy may be combined with other, classical, antimicrobials to improve clinical outcomes, and such combination therapies have now become the standard for treatment of infectious and non-infectious diseases alike^29^,^30^.

In conclusion, we have demonstrated that a biological agent with a broad spectrum activity, GST-MAM7 beads, targets bacterial adherence to reduce lethal pathogen infection *in vivo*. These findings demonstrate the potential of compounds targeting virulence traits in combating drug- resistant infections, and potentially limiting the rapid expansion of resistant bacterial populations.

## Methods

### Bacterial strains and growth conditions

*Pseudomonas aeruginosa* Xen5, a bioluminescent derivative of a blood isolate from a septic patient (strain ATCC 19660), was used throughout the experiments. The strain is resistant to carbenicillin, chloramphenicol, tetracycline and trimethoprim by Kirby-Bauer Disk Diffusion Test. *P. aeruginosa* was grown in LB broth at 37 °C under constant aeration. Bacteria was grown in log-phase to an optical density of 1.0. Bacteria suspended in LB media was introduced directly into the wound via pipette.

### Synthesis of functionalized microbeads

The adhesin MAM7 (VP1611)ΔN44 was expressed with an N-terminal GST-tag, and purified by glutathion affinity purification, gelfiltration and ion exchange chromatrography, as previously described^16^. Purified GST-MAM7ΔN44 or GST alone were chemically coupled to polystyrene microbeads, as previously described^31^, to produce inhibitor and control beads, respectively.

### Animal protocols, burn and excision model

All animal protocols were reviewed and approved by the University of Texas Southwestern Medical Center Institutional Animal Care and Use Committee. All experiments were performed in accordance with relevant guidelines and regulations.

Adult male Sprague-Dawley rats (276–300 g) were obtained from Harlan Laboratories (Houston, TX). Animals were acclimated in house for seven days after arrival at UTSW. During acclimation rats had access to ad libitum commercial rat chow and water. Rats were housed under a 12 hour light/dark cycle. After acclimation, rats were individually housed for the remainder of the experiment. Rats were deeply anesthetized with isoflurane. While under anesthesia, the area to be burned was shaved. Rats were then secured in a constructed template device. The skin exposed through the template was immersed in 100 °C water for 12 s on the back and upper sides of the body to produce full-thickness cutaneous burns over 40% total body surface area. This burn technique produced complete destruction of the underlying neural tissue. After immersion, the rats were immediately dried and fluid resuscitated. All burned animals received standard fluid resuscitation consisting of 4 ml/kg per percentage burn Lactated Ringer’s solution (~50 ml) given intraperitoneally immediately after completion of the burn injury. All burned rats were given analgesics for pain control (buprenorphine 0.5 mg/kg) immediately after injury and 12 hours post injury. After 12 hours post injury, analgesics were administered as needed.

One set of experiments was conducted to compare the efficacy of inhibitor and mock treatment in non-excised burns (n = 6 animals for the treatment and control group, respectively). In both groups, 5·10^6^ CFU of *P. aeruginosa* Xen5 was directly applied to the wound 1 day after introducing the burn, followed by topical application of suspensions containing 3·10^8^ inhibitor or control beads in saline every 24 hours.

For the burn excision model, burns were introduced as above and part of the eschar tissue corresponding to an area of approximately 4×4 cm was removed by surgical excision to the fascia. 5·10^6^ CFU of *P. aeruginosa* Xen5 was directly applied to the wound immediately following excision, followed by topical application of suspensions containing 3·10^8^ inhibitor (n = 12 animals) or control beads in saline (n = 10 animals) every 24 hours. Animals were imaged daily, using an IVIS Spectrum *In vivo* Imaging System (Perkin Elmer). Animals were sacrificed if they became moribund, or at the end of the experiment (6 days post infection). At the end of the experiment, animals were euthanized under isoflurane anesthesia. Blood and tissue were collected from each animal immediately post-mortem. Serum was aliquoted into cryovials and stored at −80 C for cytokine analysis. All experiments were performed in accordance with relevant guidelines and regulations.

### Tissue preparation and histology

A portion of the dorsal skin was fixed overnight in 10% neutral buffered formalin (NBF) and paraffin embedded. Tissue sections (5 μm) were deparaffinized and rehydrated using graded alcohol concentrations followed by H&E staining or immunofluorescence staining. For IF staining, formalin-fixed and paraffin embedded skin tissue sections were deparaffinized in xylenes and rehydrated using graded alcohol concentrations. Samples were then boiled in 10 mM citric acid for 20 min to retrieve epitopes. Sections were permeabilized with 1% Triton X-100 and blocked with 2% BSA in PBS-T. Primary antibody incubation (anti-myeloperoxidase, 1:100, Abcam ab9535) was carried out overnight at 4 °C in PBS-T + 1% BSA, and secondary antibody incubation (anti-rabbit Alexa-488, 1:500, ThermoFisher A-11034) was performed over 1 h at room temperature. Sections were finally stained for DNA using Hoechst (ThermoFisher, 33342) and mounted (Prolong gold antifade, ThermoFisher P36930). Fluorescence images were acquired on a Zeiss LSM710 confocal microscope and processed using Image J and CorelDraw X5.

### Analysis of serum cytokine levels

Serum cytokines were analyzed with Bio-Plex Pro Rat Cytokine TH1/TH2 panel according to the manufacturer’s recommended protocol with the Bio-Plex MAGPIX Multiplex Reader (Bio-Rad, Hercules, California). Data was analyzed with the Bio-Plex Data Pro software.

### Image analysis

To analyze spatial spread of infections, intensity matrices were extracted from luminescence images using MetaMorph software (Molecular Devices). Intensity data was integrated separately for each pixel row, and integrated intensity profiles were transformed to normalize midpoints of excisions to y = 0. Data sourced above and below the midpoints were expressed as positive and negative y-values, respectively.

## Additional Information

**How to cite this article**: Huebinger, R. M. *et al*. Targeting bacterial adherence inhibits multidrug-resistant *Pseudomonas aeruginosa* infection following burn injury. *Sci. Rep.* **6**, 39341; doi: 10.1038/srep39341 (2016).

**Publisher's note:** Springer Nature remains neutral with regard to jurisdictional claims in published maps and institutional affiliations.
